# Multi-Model Longevity Assays Reveal Lifespan- and Healthspan-Promoting Effects of *Bacillus subtilis* WTC019

**DOI:** 10.3390/microorganisms14020314

**Published:** 2026-01-29

**Authors:** Nan Zheng, Zhanlei Fan, Yangzhou Diao, Xiangyu Li, Yan Zhang, Bohui Shi, Jinshan Li, Shouyong Ju

**Affiliations:** 1 College of Bioengineering, Wuhan Technical University, Wuhan 430074, China; terryzn007@163.com (N.Z.); 20220021@wtc.edu.cn (Y.D.); 19371607540@163.com (X.L.); 15972373379@163.com (Y.Z.); sbh20060707@outlook.com (B.S.); 2 College of Life Science and Technology, Huazhong Agricultural University, Wuhan 430070, China; fanzhanlei@mail.hzau.edu.cn

**Keywords:** *B. subtilis*, anti-aging, lifespan, healthspan, senescence, *Caenorhabditis elegans*, human skin fibroblasts, C57BL/6J mice

## Abstract

*Bacillus subtilis* is a spore-forming probiotic with an established safety profile, yet its effects on aging-related phenotypes remain incompletely defined. Here, we assessed the lifespan- and healthspan-promoting activity of a novel strain, *B. subtilis* WTC019, using integrated invertebrate, cellular, and mammalian aging models. In *Caenorhabditis elegans*, *B. subtilis* WTC019 significantly extended lifespan, increasing median lifespan by 17.48%, 90% lifespan by 35.36%, and maximum lifespan by 19.07%, and attenuated age-associated locomotor decline. In human skin fibroblasts, *B. subtilis* WTC019 cell lysate reduced senescence-associated β-galactosidase activity by approximately 34% and altered cell cycle distribution consistent with delayed cellular senescence. Moreover, dietary supplementation with *B. subtilis* WTC019 significantly prolonged lifespan in C57BL/6J mice, with median lifespan increases of 5.97% in females and 6.05% in males. Together, these results demonstrate that *B. subtilis* WTC019 promotes lifespan- and healthspan-associated phenotypes across multiple aging models, supporting its potential as a probiotic candidate for healthy aging interventions.

## 1. Introduction

Aging is a complex biological process characterized by progressive decline in cellular function and systemic homeostasis, ultimately resulting in increased susceptibility to disease and death [1].

*Bacillus subtilis* is a Gram-positive spore-forming bacterium and commonly found in soil and fermented foods. It has emerged as a major workhorse in biotechnology, owing to its ability to secrete large quantities of proteins and produce a broad range of commercially valuable compounds [2]. As a widely used probiotic, *B. subtilis* exerts multiple beneficial effects, including regulating gut microbiota [3], improving animal growth performance [4], enhancing host immunity [5], and offering advantages of short fermentation cycles and cost-effectiveness [6]. In recent years, several *B. subtilis* strains have been shown to exhibit lifespan-extending effects on *C. elegans* [7,8]. These findings suggest that *B. subtilis* may be a functionally versatile probiotic with anti-aging potential, yet its capacity to modulate aging processes has been underexplored.

In our previous work [9], we isolated a new strain termed *B. subtilis* WTC019, whose biological effects have not been thoroughly characterized. Thus, in this study, we sought to evaluate the anti-aging potential of *B. subtilis* WTC019 using different aging models. First, we employed *Caenorhabditis elegans*, a well-established invertebrate model for high-throughput longevity screening, to conduct preliminary assessments on lifespan and healthspan [7,10]. Worms fed with *B. subtilis* WTC019 exhibited a significantly enhanced lifespan and healthspan compared to control worms fed the standard laboratory bacterial diet *E. coli* OP50. We next tested *B. subtilis* WTC019 in human skin fibroblasts (HSFs), a widely used model for mammalian cellular aging; treatment of HSFs with *B. subtilis* WTC019 lysate led to a significant reduction in senescence-associated β-galactosidase activity by balancing cell cycle distribution [11,12]. Thereafter, further validation of its life-extending activity in mammalian organisms was conducted using C57BL/6J mice [13,14]. Longitudinal survival monitoring showed that mice receiving *B. subtilis* WTC019 have a significantly longer lifespan than control mice fed with chow diet.

Collectively, these results demonstrate that *B. subtilis* WTC019 exhibits consistent lifespan-promoting effects across three distinct aging models. This multi-model validation confirms the strain’s longevity-promoting function and supports its potential as a probiotic candidate for aging intervention.

## 2. Materials and Methods

### 2.1. Bacterial Strains and Culture

*B. subtilis* strain WTC019 was previously isolated and identified in our laboratory [9]. WTC019 was isolated from straw-compost soil in Wuhan, Hubei Province. The physiological and biochemical profiles of WTC019 were consistent with those of the standard strain. 16S rRNA gene analysis combined with physiological and biochemical identification confirmed that WTC019 is a strain of *Bacillus subtilis*.

The strain was cultured in Luria–Bertani (LB) medium at 200 rpm until the stationary phase was reached. Cells were subsequently centrifuged at 12,000 rpm for 5 min. The cell pellet was washed once in 10 mM phosphate buffer (pH 6.8). The resulting suspension was freeze-dried to obtain a lyophilized powder, which was used for *C. elegans* and mouse lifespan assays. Colony-forming units (CFUs) were determined by serial dilution and plate counting on LB agar plates.

### 2.2. Bacterial Cell Lysate Preparation

*B. subtilis* WTC019 cells were harvested according to previous methods [15]. Harvested cells were washed once with 10 mM phosphate buffer (PBS, pH 6.5) and subsequently resuspended in 10 mL of the same buffer. Cell disruption was performed on ice using an ultrasonic homogenizer (SCIENTZ-IID, Ningbo Scientz Biotechnology, Ningbo, China) with the following parameters: 300 W power output, 30% duty cycle (10 s on/20 s off), and total processing time of 30 min. Following sonication, the cell suspension was filtered through a 0.22 μm sterile filter to remove intact cells. The resulting filtrate was centrifuged at 30,000× *g* for 60 min at 4 °C to pellet insoluble cell debris. The *B. subtilis* WTC019 cell lysate was collected and stored at −80 °C for β-galactosidase assay and cell cycle analysis.

### 2.3. Caenorhabditis elegans Strains and Culture

The wild-type *C. elegans* strain N2 was obtained from the CGC (Caenorhabditis Genetic Centre, Minneapolis, MN, USA) and maintained on standard nematode growth medium (NGM) plates seeded with *E. coli* OP50 at 20 °C [16]. For synchronization, gravid adult nematodes were treated with Clorox solution (5% NaOCl/1M NaOH = 2:1, *v/v*) for 10 min, and then the nematode suspensions were centrifuged at 2200 rpm for 2 min. The collected embryos were maintained on new NGM agar plates with *E. coli* OP50 at 20 °C.

### 2.4. C. elegans Lifespan and Locomotor Function Assay

Lifespan assays were performed at 20 °C on NGM plates as described before [17]. Bacterial suspensions (*E. coli* OP50 or *B. subtilis* WTC019) were fully spread on the NGM plate to prevent worms from avoiding or escaping the bacterial lawn. Approximately 60 L4-stage worms were incubated on NGM plate seeded with 1 × 10^9^ *E.coli* OP50 or 2 × 10^8^ CFU/mL *B. subtilis* WTC019. *E.coli* OP50 was supplied at slightly higher levels than *Bacillus subtilis* WTC019 to ensure sufficient food availability without impairing feeding behavior. Every plate contained 0.05 mg/mL of 5-fluorodeoxyuridine (FUDR) to prevent eggs from hatching. Each experiment was independently replicated at least three times. Survival was recorded daily. Worms that failed to respond to gentle prodding were scored as dead. The surviving worms on each plate were counted at 20 °C every 12 h. Statistical analyses were assessed by Kaplan–Meier survival analysis followed by a log-rank test. For locomotor function assessment, head thrash frequency was measured on days 5, 10, 15, 20, and 25 of adulthood. A head thrash was defined as a change in the direction of bending at the midpoint of the body. Each measurement was conducted over a 30 s interval in M9 buffer.

### 2.5. HSF Cell Culture and β-Galactosidase Assay

The human skin fibroblast (HSF) cells obtained from the China Center for Type Culture Collection (CCTCC) were cultured in Dulbecco’s modified Eagle’s medium (DMEM, Gibco, Waltham, MA, USA) supplemented with 10% fetal bovine serum (FBS, Hyclone, Logan, UT, USA) and 1% penicillin/streptomycin (Gibco), at 37 °C in a humidified incubator containing 5% CO_2_ [11]. All cells were washed twice. For the β-galactosidase assay, approximately 5 × 10^5^ HSF cells/well were plated in 6-well plates and allowed to attach for 24 h, followed by treatment with 50 μg/mL sonicated bacteria extracts of *B. subtilis* WTC019 or *E.coli* OP50 for 48 h. β-galactosidase activity was detected using a senescence-associated β-galactosidase staining kit (CS0030, Merk, Darmstadt, Germany). The percentage of SA-β-Gal-positive cells was calculated from at least five random microscopic fields per sample.

### 2.6. Cell Cycle Analysis

HSF cells were plated in 6-well plates and allowed to attach 24 h, with treatment of *B. subtilis* WTC019 cell lysate or Control. After treatment with the *B. subtilis* WTC019 cell lysate, HSF cells were harvested, fixed in 70% ethanol at 4 °C overnight and washed twice with ice-cold PBS before staining. Cells were stained with propidium iodide (PI) solution containing RNase A. Cell cycle distribution was analyzed by flow cytometry (FACSCanto II, BD, Franklin Lakes, NJ, USA) with 10,000 events per sample, and the proportions of cells in G_0_/G_1_, S, and G_2_/M phases were determined using FlowJo v10.6 software [18].

### 2.7. Cell Viability Assay

Cell viability was detected by the colorimetric analysis of blue tetrazolium bromide thiazolyl (MTT). After treatment with bacteria lysate, 20 μL of MTT (a final concentration of 0.5 mg/mL) was incubated for 4 h in a humidified incubator containing 5% CO_2_ at 37 °C. Then, 100 μL of dimethyl sulfoxide (DMSO) was added to the media and the absorbance at 570 nm was detected using a Microplate reader (Thermo Fisher Scientific, Shanghai, China).

### 2.8. Mice and Dietary Regimens

Male and female C57BL/6J mice were purchased at 14 months of age from Shulaibao Biotechnology Co., Ltd., China. All mice were initially maintained on a standard irradiated chow diet (101WC-008; Jiangsu Xietong Pharmaceutical Bio-Engineering Co., Nanjing, China) containing 18% crude protein and 6% crude fat. Animals were housed in a specific pathogen-free (SPF) facility under controlled environmental conditions: temperature at 22 ± 1 °C, relative humidity at 50–70%, and a 12 h light/dark cycle. Throughout the study, mice had ad libitum access to the assigned diet and autoclaved drinking water.

### 2.9. Mice Lifespan Assay

Mice lifespan assays were performed as described before [13,19]. Mice were aged to 18 months before the initiation of experimental treatments. Two independent cohorts (stratified by sex, with both female and male mice included) of 18-month-old C57BL/6J mice were used. Within each cohort, mice were randomly allocated to one of two experimental groups, with approximately 40 mice per group. The control group was fed the standard irradiated chow diet. The *B. subtilis* (Bs) treatment group was fed the same standard irradiated chow diet supplemented with lyophilized cells of *B. subtilis* WTC019 at a final concentration of 1 × 10^10^ colony-forming units (CFU) per kilogram of feed. Mice were housed undisturbed except for daily health monitoring. The date of death was recorded for any mice found deceased. No mice were euthanized for humane endpoints (e.g., severe fighting wounds, moribundity) or excluded due to technical issues (e.g., escape, accidental injury). All experimental procedures, including animal care and treatment, were approved by the Animal Care and Use Committee (ACUC) of Wuhan Technical University.

### 2.10. Statistical Analysis

All experiments were conducted in triplicate unless otherwise stated. Survival data were analyzed using Kaplan–Meier survival curves, and statistical significance was assessed by the log-rank (Mantel–Cox) test. Other data were expressed as the mean ± standard deviation (SD). Statistical differences between groups were analyzed using a one-way ANOVA test. A *p*-value of < 0.05 was considered statistically significant.

## 3. Results

### 3.1. B. subtilis WTC019 Extends Lifespan in C. elegans

*B. subtilis* WTC019 was isolated in our previous work [9] (Supplementary Data, Figures S1 and S2). To preliminarily investigate the anti-aging activity of *B. subtilis* WTC019, the well-established longevity model *C. elegans* was used to test its lifespan-extending effects. Lifespan assays were conducted with 60 wild-type *C. elegans* per group.

**Figure 1:**
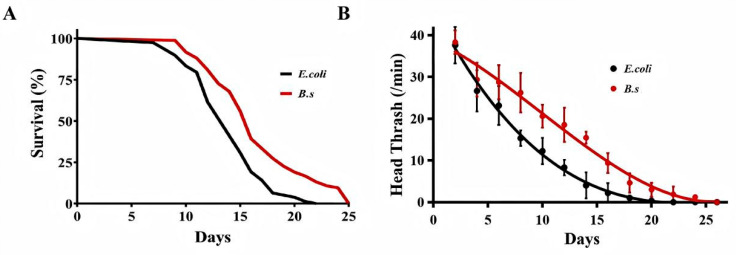
Effect of *B. subtilis* WTC019 on of the healthspan extension of *C. elegans*. (**A**) The lifespan of worms fed with *B. subtilis* WTC019 was significantly longer than that of worms fed with the standard diet *E. coli* OP50. *n* = 100, *p* = 0.006 versus control. Survival data were analyzed using Kaplan–Meier survival curves, and statistical significance was assessed by the log-rank (Mantel–Cox) test. (**B**) *B. subtilis* WTC019 alleviated the age-related decline in head thrash frequency. *n* = 20, mean ± SD, *p* < 0.05. Different superscript letters denote statistically significant differences.

Lifespan analysis was conducted on 60 wild-type *C. elegans* N2 (Figure 1A, Table 1). The control group fed with *E. coli* OP50 had a median lifespan (age at 50% mortality) of 14.3 days, 90% lifespan (age at 90% mortality) of 17.4 days, and maximum lifespan (age at 100% mortality) of 21.5 days (Table 1). In contrast, *C. elegans* fed with *B. subtilis* WTC019 (B.s group) showed significant lifespan extensions relative to the control group. The median lifespan increased by 17.48% to 16.8 days, 90% lifespan by 35.36% to 23.6 days, and maximum lifespan by 19.07% to 25.6 days (Figure 1A, Table 1). These results demonstrated that *B. subtilis* WTC019 promotes longevity in *C. elegans*.

### 3.2. B. subtilis WTC019 Enhances Locomotor Activity in C. elegans

To assess effects on age-related locomotor decline, the head thrash frequency of *C. elegans* was measured. *B. subtilis* WTC019 significantly increased head thrash frequency relative to the control at all time points (Figure 1B), indicating mitigation of age-associated movement capacity loss. The greatest difference in locomotor activity between groups was observed on day 14. The head thrash frequency was 4.39 ± 3.82 in the control group versus 16.55 ± 1.62 in the *B. subtilis* WTC019-treated group. Collectively, these findings demonstrate that *B. subtilis* WTC019 effectively improves healthspan in *C. elegans*.

### 3.3. B. subtilis WTC019 Cell Lysate Inhibits Senescence in Human Skin Fibroblasts Cells

**Figure 2:**
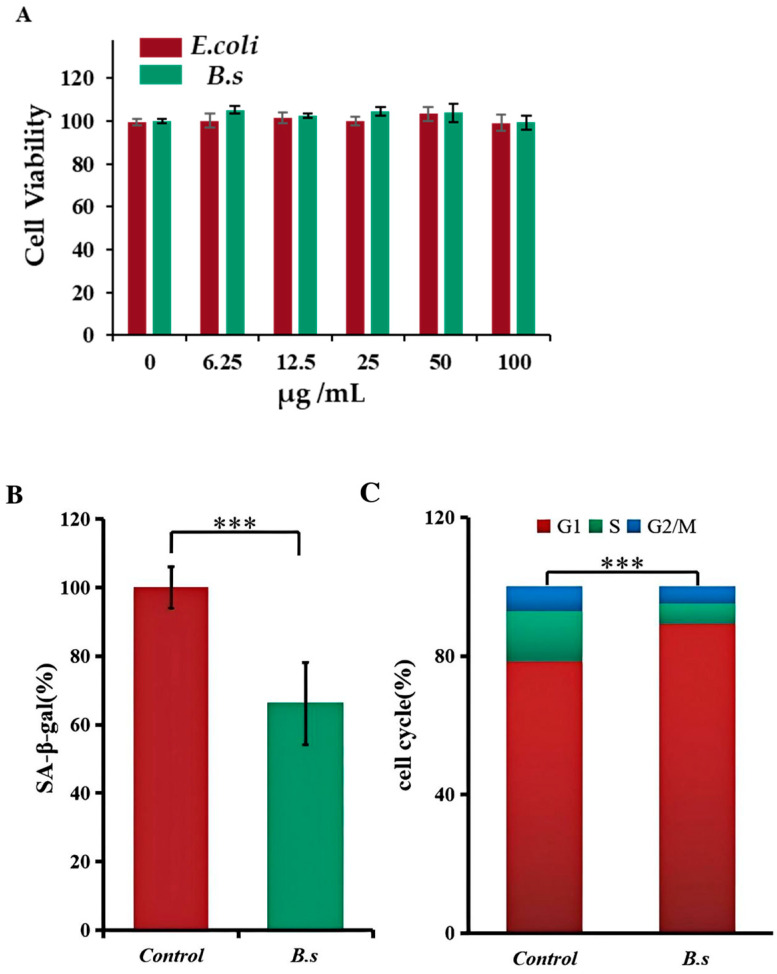
*B. subtilis* WTC019 cell lysate inhibits senescence in human skin fibroblasts (HSFs). (**A**) Indicated concentration of bacteria lysate did not significantly inhibit HSF cell viability in the MTT test. Mean ± SD, *n* = 6, *p* > 0.05, one-way ANOVA. (**B**) *B. subtilis* WTC019 extract reduces β-galactosidase activity in HSF cells. Mean ± SD, *n* = 6, *** means *p* < 0.001, one-way ANOVA. (**C**) HSF cells were stained with PI and then HSF cell cycle were analyzed by flow cytometry. *B. subtilis* WTC019 extract inhibits HSF cell proportion in the S and G2/M phases. Mean ± SD, *n* = 6, *** means *p* < 0.001, one-way ANOVA.

The degree of senescence in animal cells can be quantitatively analyzed by detecting β-galactosidase activity and the proportion of cells in each cell cycle phase [11]. To avoid confounding effects of bacterial overgrowth in cell culture, *B. subtilis* WTC019 cell lysate rather than live bacteria was used for the following test. We first proved that less than 100 μg/mL sonicated extracts of *B. subtilis* WTC019 or *E.coli* OP50 exhibit no significant cytotoxicity to HSF cells (Figure 2A). Then, we employed the dose of 50 μg/mL bacteria extracts of *B. subtilis* WTC019 or *E.coli* OP50 for treatment. Bacteria extracts were incubated with human skin fibroblast (HSF) cells for 48 h before analysis. β-galactosidase activity in HSF cells is shown in Figure 2B. The addition of *B. subtilis* WTC019 cell lysate resulted in a 34.31% ± 12% decrease in β-galactosidase activity within the cells compared to control.

In the meantime, we employed flow cytometry analysis to further investigate the effect of *B. subtilis* WTC019 cell lysate on HSF cell cycle distribution, as alterations in specific cell cycle phases are closely associated with cellular senescence [18]. As shown in Figure 2C and Supplementary Figure S3, the proportion of cells for the control group was 78.3% ± 2.4% in G1 phase, 15.1% ± 1.7% in S phase and 6.6% ± 1.1% in G2/M phase, whereas for the group treated with *B. subtilis* WTC019 cell lysate, the proportion of cells shift to 89.9% ± 2.5% in G1 phase, 6.2% ± 1.3% in S phase and 3.9% ± 0.9% in G2/M phase. Compared to the control group, the addition of *B. subtilis* WTC019 cell lysate led to a substantial increase in G1 cell proportion, along with a decrease in S and G2/M cell proportion. This demonstrates that the addition of *B. subtilis* WTC019 extract decelerates HSF cells from entering the proliferative phases (S and G2/M), thereby prolong the cell cycle of HSF cells.

### 3.4. B. subtilis WTC019 Extends Lifespan of C57BL/6 J Mice

**Figure 3:**
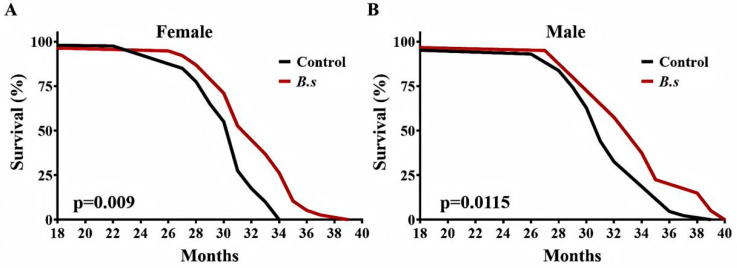
*B. subtilis* WTC019 increases longevity of C57BL/6J mice. (**A**) Survival curves for female mice treated with daily intake of either with *B. subtilis* WTC019 (1 × 10^10^ CFU/kg) (red line, *n* = 38) or untreated counterparts (black line, *n* = 40). (*p* = 0.009, Log-rank (Mantel–Cox) test). (**B**) Kapla–Meier survival curves for male mice treated with daily intake of either with *B. subtilis* WTC019 (1 × 10^10^ CFU/kg) (red line, *n* = 40) or untreated counterparts (black line, *n* = 43). *p* = 0.0115, log-rank test.

Mice were fed regular chow diet to 18 months of age and then switched to a diet supplemented with *B. subtilis* WTC019 (1 × 10^10^ CFU per kilogram of feed). Supplementation with *B. subtilis* WTC019 significantly prolonged survival in both female and male mouse cohorts compared to age-matched control mice (Figure 3A,B, Table 2). In the cohort of female mice, median, 90% and 100% survival ages were significantly extended by 5.97% and 7.80% and 9.27%, respectively (Figure 3A, Table 2). In the cohort of male mice, median, 90% and 100% survival ages were significantly extended by 6.05%, 10.95% and 8.14%, respectively (Figure 3B, Table 2). Notably, no significant differences in daily food or water intake were observed between the *B. subtilis* WTC019-supplemented group and the control group. Collectively, these results demonstrate that dietary supplementation with *B. subtilis* WTC019 induces a significant extension of lifespan in mice.

## 4. Discussion

Spore-forming *B. subtilis* has emerged as an increasingly promising probiotic, primarily attributed to its superior shelf stability and enhanced capacity to survive transit through the human gastrointestinal tract relative to other bacterial species [2]. While the viability, safety, and tolerability of specific *B. subtilis* strains have been evaluated in relevant human cohorts, the full spectrum of their physiological benefits remains insufficiently characterized, particularly in the context of aging [3].

Existing research has highlighted diverse probiotic functional roles of *B. subtilis* across model systems and human cohorts, including in *C. elegans*, mice, and healthy or elderly humans. *B. subtilis* has been shown to exhibit the strongest lifespan-extending effect on *C. elegans* through down-regulation of the insulin-like signal pathway [20] and subsequent studies have revealed that microbial nitric oxide (NO) synthesis and detoxification are critical for mediating the anti-aging and enhancing the locomotor activity effects of *B. subtilis* [7,21]. In addition, supplementation of *B. subtilis* DE111 induced favorable changes in lipid profiles and improved endothelial function in healthy adults [22]. Moreover, *B. subtilis* has been reported to assist the immune system through multiple mechanisms. Periodic administration of *B. subtilis* CU1 increases fecal and salivary secretory IgA levels in older individuals [23]. Another piece of research also revealed that an inactivated *B. subtilis* could inhibit *Porphyromonas gingivalis*-induced gingival inflammation in mice [24].

In this study, the lifespan- and healthspan-improving effects of *B. subtilis* were systematically confirmed across three distinct aging models (*C. elegans*, human skin fibroblasts, and C57BL/6J mice) to preliminarily evaluate its anti-aging potential. The cellular senescence assays using a human skin fibroblast model showed a reduced proportion of senescent cells and down-regulation of cell proliferation. This indicated that *B. subtilis* WTC019 can decelerate cell cycle progression and thereby improves lifespan at the cellular level.

Furthermore, the *C. elegans* and mice assays provided organismal-level validation on the basis of these cellular findings. Supplementation with *B. subtilis* WTC019 extended lifespan and mitigated the decline in locomotor performance typically associated with aging. These findings are in line with the report that another strain of *B. subtilis* delays age-associated phenotypes and behavioral impairment in the Alzheimer’s disease model *C. elegans* [25]. Our data also proved the efficacy of *B. subtilis* WTC019 for life extension as it increased median lifespan of *C. elegans* by 17.48%, 90% lifespan by 35.36% and maximum lifespan by 19.07% (Figure 1A, Table 1). In comparison, it was reported that *B. subtilis* 168 can increase mean lifespan of *C. elegans* by 21% and maximum lifespan by 11%; *B. subtilis* TO-A can increase mean lifespan of *C. elegans* by 35% and maximum lifespan by 26% [7]. These results provide a comparative baseline for WTC019′s efficacy, which has not yet been compared with any well-documented anti-aging compound as the positive control. In spite of the nuances of experimentation, these *B. subtilis* strains exhibit similar capacity in lifespan-promoting efficacy in *C. elegans*. Moreover, it was reported that like the effect showed by *B. subtilis* WTC019, various laboratory *B. subtilis* strains, such as 168, JH642, NCIB 3610 and PXN21, shared a similar protective effect against α-synuclein aggregation [8]. Thus, anti-aging might be a general property for *B. subtilis* strains. However, additional research is necessary to explore the anti-aging effects of *B. subtilis* strains at multiple organism-levels.

The observed lifespan- and healthspan-associated effects of *B. subtilis* WTC019 suggest potential relevance for probiotic-based approaches to healthy aging. *B. subtilis* strains are widely used as commercial probiotics due to their spore-forming capacity, formulation stability, and established history of safe use [2]. However, probiotic properties are strain-specific, and some *B. subtilis* strains may harbor toxin genes or antibiotic resistance determinants, necessitating comprehensive strain-level safety evaluation prior to translational application [26]. In this study, much as the *B. subtilis* WTC019′s efficacy is validated in the longevity assay in *C. elegans* and the mouse model, the optimal effective dose and potential toxicity at higher doses should be further determined before further clinical trials and potential translational applications.

Above all, this study merely provides a phenotypic screening and validation of *B. subtilis* WTC019 across models and the underlying mechanisms require future investigation. We hypothesize that the mechanism of WTC019′s lifespan-promoting effect may resemble that of other strains of *B. subtilis*, as in our work of strain characterization, we found it exhibits numerous similarities with standard strain *B. subtilis* 168 regarding physiological and biochemical phenotype (Supplementary Data, Figure S1, Table S1). Thus, future mechanistic research should be conducted using transcriptomic analysis in different models [27] and targeted pathway assays including insulin/IGF signaling and microbial nitric oxide metabolism [20,28]. In addition, the classic stress response signaling involving DAF16 should be tested as it is the classic anti-aging target in *C. elegans* [29]. Notably, as *B. subtilis* spores and vegetative cells both play a protective role in the aging process, through distinct independent mechanisms [8], it might be necessary to demonstrate sporulation efficiency and spore purity in further mechanistic research.

In conclusion, our findings demonstrate that *B. subtilis* WTC019 improves healthspan-associated phenotypes and extends lifespan across multiple aging models, supporting its potential application in promoting healthy aging.

## Figures and Tables

**Table 1:** Survival statistics for the effects of *B. subtilis* WTC019 in *C. elegans.*

Group	Age (Days) at 50% Mortality	Percentage Survival Increase (Bs vs. CK)	Age (Days) at 90% Mortality	Percentage Survival Increase (Bs vs. CK)	Age (Days) at 100% Mortality	Percentage Survival Increase (Bs vs. CK)	One-Way ANOVA *p*-Value
CK	14.3		17.4		21.5		
B.s	16.8	17.48%	23.6	35.36%	25.6	19.07%	*p* < 0.05

**Table 2:** Survival statistics for the effects of *B. subtilis* WTC019 in C57BL/6J mice.

Group	Age (Days) at 50% Mortality	Percentage Survival Increase (Bs vs. CK)	Age (Days) at 90% Mortality	Percentage Survival Increase (Bs vs. CK)	Age (Days) at 100% Mortality	Percentage Survival Increase (Bs vs. CK)	Log-Rank *p*-Value
Female							
CK	904		990		1014		
*B.s*	958	5.97%	1069	7.80%	1108	9.27%	*p* < 0.01
male							
CK	925		995		1105		
*B.s*	981	6.05%	1104	10.95%	1195	8.14%	*p* < 0.05

## Data Availability

The original contributions presented in this study are included in the article/Supplementary Materials. Further inquiries can be directed to the corresponding authors.

## Supplementary Materials

The following supporting information can be downloaded at: https://www.mdpi.com/article/10.3390/microorganisms14020314/s1, Figure S1: The phylogenetic tree based on 16S rDNA; Figure S2: PCR products electrophoresis of 16S rRNA gene; Figure S3: *B. subtilis* WTC019 extract inhibit HSF cell proportion in S and G2/M phase. HSF cell cycle was detected using PI staining followed by flow cytometer. *p* < 0.01. *n* = 6, mean ± SD, one-way ANOVA; Table S1: Physiological and biochemical identification of *Bacillus subtilis* WTC019.
