# Supplementary material for: Multi-Model Longevity Assays Reveal Lifespan- and Healthspan-Promoting Effects of Bacillus subtilis WTC019
Source: Microorganisms. 2026 Jan 29;14(2):314. doi: 10.3390/microorganisms14020314 (PMC12943744; doi:10.3390/microorganisms14020314)
Supplement: Supplementary file 1 [file microorganisms-14-00314-s001.zip › microorganisms-4092775-supplementary.pdf]

Supplementary data

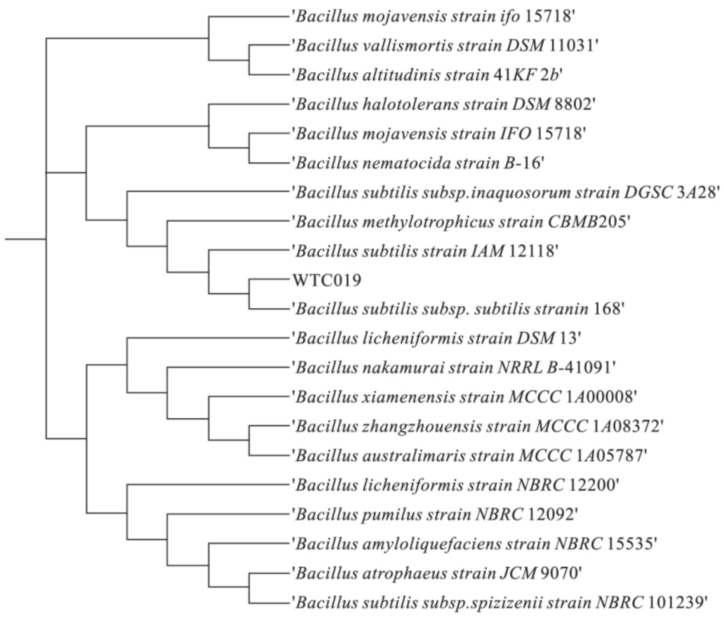

Figure S1. The phylogenetic tree based on 16S rDNA

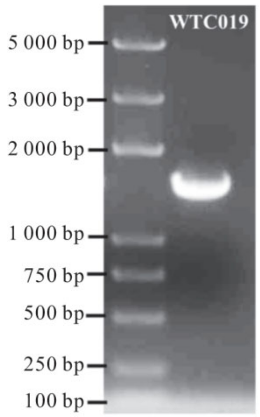

Figure S2. PCR products electrophoresis of 16S rRNA gene

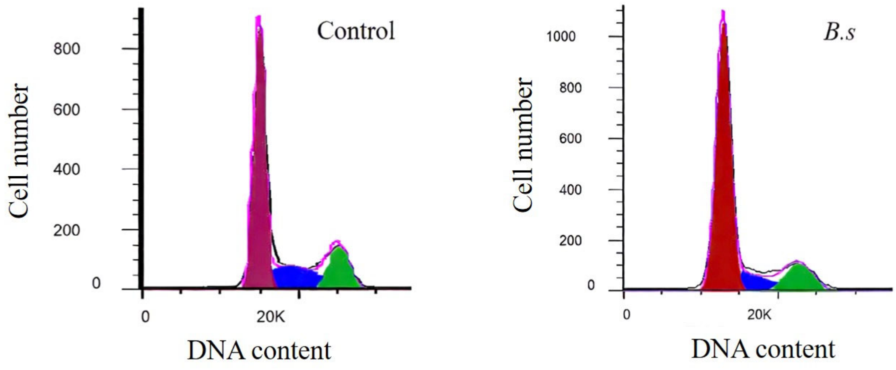

Figure S3. *B. subtilis* WTC019 extract inhibit HSF cell proportion in S and G2/M phase. HSF cell cycle was detected using PI staining followed by flow cytometer. $p<0.01$. n= 6, mean ± SD, one-way ANOVA

Physicochemical indicators	*B. subtilis* WTC019	*B. subtilis* 168
Gram staining	+	+
Catalase test	+	+
Methyl Red	+	+
Milk	+	+
Gelatin	+	+
Lipase (tween 60)	+	+
Glucose	+	+
Sucrose	-	-
Myo-inositol	-	-
D-xylose	+	+
D-fructose	-	-
Cellobiose	-	-
L-arabinose	+	+
D-galactose	-	-
Lactose	+	+
Maltose	+	+
Raffinose	+	+
Starch	+	+
Mannitol	+	+
Xylitol	-	-
Indole	-	-
Nitrate reduction	+	+
Nitrite Reduction	+	+
Citrate	+	+
Acetate Oxidation	+	+

+, positive; –, negative.

Table S1. Physiological and biochemical identification of *Bacillus subtilis* WTC019
